# Effects of Ultrasound Treatments on Tenderness and In Vitro Protein Digestibility of New Zealand Abalone, *Haliotis iris*

**DOI:** 10.3390/foods9081122

**Published:** 2020-08-14

**Authors:** Norma Cecille Bagarinao, Lovedeep Kaur, Mike Boland

**Affiliations:** 1 School of Food and Advanced Technology, Massey University, Palmerston North 4442, New Zealand; nbagarinaodost8@gmail.com; 2 Riddet Institute, Palmerston North 4442, New Zealand; M.Boland@massey.ac.nz

**Keywords:** abalone, pāua, *Haliotis iris*, ultrasound, actinidin, texture, in vitro protein digestibility

## Abstract

Canned pāua, *Haliotis iris*, is a premium New Zealand product that is exported to Asia. The objective of this research was to investigate the effects of ultrasound treatments on pāua texture, microstructure and in vitro protein digestibility. Whole pāua meat was ultrasound-treated (20 kHz, 464 ± 9 W) for 5 min in water (with or without subsequent soaking in water at 4 °C for 24 h) or ultrasound-treated in 1% actinidin enzyme solution. Post-treatment cooking of canned pāua was done in a water retort at 116 °C for 30 min. All ultrasound-treated cooked pāua yielded lower slice shear force values (SSFV) than untreated canned and cooked samples. The lowest SSFV was attained when ultrasound treatment in water was followed by soaking at 4 °C for 24 h. The increased tenderness of ultrasound-treated pāua could be linked to disintegration of myofibers and formation of gaps between myofibers, as observed through histological analysis and transmission electron microscopy. Collagenous fragmentation was also observed, particularly in pāua ultrasonicated in enzyme solution. Raw pāua was found to be more digestible in terms of free amino N released during in vitro digestion than all cooked samples. However, cooked ultrasound pre-treated pāua was more digestible than the control cooked sample.

## 1. Introduction

Pāua, the Māori name for an abalone species found in New Zealand, are highly valued by Māori and are recognised as a *taonga*, or treasure. Their colourful shell was traditionally used to decorate wood carvings, and today they are used for making iconic jewellery. The flesh is considered a delicacy. There is a large commercial market for their flesh, primarily in Asia. It is a highly priced New Zealand export product that is sold live, fresh or in processed form. In the five years to 2015, on average, the total value of pāua caught was $56.9 million [1]. The most important commercial New Zealand species is *Haliotis iris* or the black foot pāua, which is among the largest abalone species in the world.

Meat toughness and chewiness are common problems associated with abalone species in many parts of the world [2,3,4,5]. There is a wide range of literature available on the use of different processing techniques for tenderising abalone, mostly for Asian abalone species. Several articles focus on the effect of thermal [3,6,7,8] and high-pressure processing on abalone [9,10]. However, limited studies have been done on *Haliotis iris*, and these have been mainly focused on the effects of diets [11,12], habitats and physiological and environmental conditions [13] on the quality of the raw abalone meat. 

Studies by Zhu et al. 2018 [14] have shown the potential of the proteolytic enzyme from kiwifruit, actinidin, and its combination with sous-vide cooking in tenderising tough beef cuts. Positive results on red meat texture have also been reported for non-thermal processing methods, such as ultrasound and marinating [15]. Changes in the muscle structure, induced by processing, have been reported to be determinants of product texture and digestibility [14]. Cepero-Betancourt et al. [16] examined the protein digestibility, in rats, of dried abalone, *Haliotis rufescens*, pre-treated using high pressure processing. From the nutritional parameters, which included the amount of essential amino acids (EAA), protein efficiency ratio (PER), true digestibility (TD), net protein ratio (NPR) and protein digestibility corrected amino acid score (PDCAAS), the control untreated abalone sample exhibited high-quality nutritional protein [16]. 

In the current study, it was hypothesised that the structural changes induced by ultrasound would not only affect texture, but would also affect the protein digestibility of canned pāua. Addition of actinidin enzyme during ultrasound treatment might allow penetration of the enzyme into the inner meat layers that could lead to further tenderisation of pāua meat. Therefore, this study was focused on investigating the effects of ultrasound alone or along with actinidin enzyme treatment on the tenderness, in vitro protein digestibility and microstructure of canned meat from *Haliotis iris*.

## 2. Materials and Methods

### 2.1. Materials

**Figure 1:**
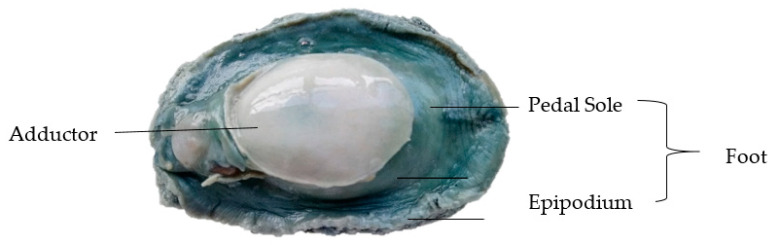
Raw untreated pāua.

Deshelled and gutted whole wild pāua with an average weight of 170 ± 20 g were kindly provided by Prepared Foods Limited (Palmerston North, New Zealand) and a local fishmonger (Deli-ca-sea, Palmerston North, New Zealand). Samples were washed, vacuum-packed and stored at 4 °C for at least 26 h post-mortem to allow post-rigor to set in before processing. A photograph of the sample segmented into three parts—adductor, pedal sole and epipodium (both parts of the foot) [17]—is shown in Figure 1. Any mention of the foot in the following sections refers to the pedal sole, unless otherwise stated. Commercial canned pāua samples were also kindly provided by Prepared Foods Limited. 

Commercial actinidin enzyme powder (Actazin™) was bought from Anagenix Ltd. (Auckland, New Zealand). All the chemicals and reagents used in the experiments and analyses were of analytical grade.

### 2.2. Sample Treatments

Samples were subjected to multiple preliminary treatments and the treatments that led to texture improvement in terms of slice shear force values (SSFV, measured as explained in Section 2.3.6) without adversely affecting the sample appearance were selected for the experiments presented in this study. The selected treatments that are discussed in this study include: (1) ultrasound in water for 5 min, (2) ultrasound in water for 5 min followed by 24 h soaking in water at 4 °C, (3) ultrasound in 1% actinidin extract solution for 5 min, (4) soaking in water for 24 h at 4 °C, and (5) untreated control. All samples were immediately vacuum tumbled for 30 min, then put in a can (74 mm × 119 mm) and filled with hot Milli-Q water (45–55 °C). After this, the samples were cooked in water retort at 116 °C for 30 min. 

The duration for all ultrasound treatments was 5 min, which gave the lowest SSFV during preliminary experiments. Whole samples, one at a time, were placed in a one-litre glass beaker with 700–800 mL of Milli-Q water or 1% (*w*/*v*) enzyme solution in water. The enzyme solution was prepared fresh on the day of the experiment by dissolving actinidin powder in Milli-Q water at 4 °C. The beaker was placed inside a circulating cold-water bath (Julabo F25, Julabo GmbH, Seelbach, Germany) maintained at 0–2 °C [18]. The ultrasound sonotrode was immersed in the beaker with the tip about 1 cm above the pāua. Ultrasound (UIP1000hd, Heilscher Ultrasonics GmbH, Tetlow, Germany) was carried out at an average power output of 464 ± 9 Watts, 100% amplitude and at 20 kHz frequency.

For soaking, samples were soaked in Milli-Q water under refrigerated conditions (4 °C) for 24 h. Pāua were placed inside a covered container with 500 mL of water, enough to cover the sample, one pāua in each container. 

### 2.3. Analyses of Control and Treated Pāua Samples

Raw treated samples were analysed for any changes in protein denaturation profiles, pH and muscle fibre structure. Cooked samples were evaluated for pH, SSFV and muscle fibre structure. The in vitro protein digestibility of (1) raw untreated control, (2) cooked control and (3) sample treated in ultrasound in water for 5 min followed by soaking for 24 h in water and cooking, were determined. Sample no. 3 was chosen for the protein digestibility analysis since the treatment gave the lowest SSFV as discussed in Section 3.1. 

#### 2.3.1. pH Measurements

The pH of the raw and cooked samples was determined using a pH meter (Orion 3 Star, Thermo Electron Corporation, Waltham, MA, USA) by inserting a spear tip probe (Sensorex, Garden Grove, CA, USA) and thermocouple into the centre part of the pāua. The probe was rinsed with reverse osmosis water between samples.

#### 2.3.2. Differential Scanning Calorimetry (DSC)

The method of Chian et al. [19] was adopted for the determination of the protein denaturation in raw control and ultrasound-treated samples using a DSC (Q2000, TA instruments, New Castle, DE, USA). Samples from the pāua foot were comminuted and homogenised. Approximately 15 mg of comminuted and homogenised samples were weighed into DSC pans, sealed and heated together with an empty pan that was used as a reference. The temperature range was between 20–100 °C, increasing at a rate of 2 °C/min. The resulting thermal curves were analysed using the TA Universal Analysis Software (TA Instruments, New Castle, DE, USA). 

#### 2.3.3. Cook Loss (%)

Cook loss was determined as the difference in weight between the cooked and uncooked samples, as expressed in Equation (1):

(1) $$\%\;\mathrm{Cook}\;\mathrm{Loss}=\;\frac{\left(W_{A}-W_{B}\right)}{W_{A}}\;\times \;100$$

where *W_A_* is the weight of meat before cooking, and *W_B_* is the weight of meat after cooking. 

#### 2.3.4. Microstructure Analysis Using Verhoeff-Van Gieson Staining (VVG) 

The VVG staining procedure was adopted from the method of Culling [20] with some modifications. Images of the stained cells appeared red–dark brown for collagen fibres and yellow–light brown for myofibrils when viewed under a light microscope. 

Pre-treatment. Raw and cooked treated samples from the centre part of the foot were cut into 5 mm × 5 mm sections and frozen in optimal cutting temperature compound (OCT) using an aluminium brick previously stored for at least 24 h at −80 °C. Cut sections were embedded into stainless steel moulds with OCT. Moulds were set on top of the pre-frozen aluminium brick inside a Styrofoam box, covered and allowed to be frozen for 10 min. Embedded sections were sliced into 5 µm sections parallel to the fibre direction using a cryostat microtome (Leica, Wetzlar, Germany, Jung CM1800) at −16 °C, before mounting onto glass slides. 

VVG Staining. Sections were fixed with methanol for 6 min, then stained with freshly made Verhoeff’s solution (5% alcoholic haematoxylin:10% ferric chloride:10% Verhoeff’s iodine solution at a ratio of 2.5:1:1) for 15 min. Stained sections were then rinsed with 2–3 changes of RO water followed by differentiation with 2% ferric chloride for 90 sec. After this, slides were washed in RO water and rinsed with 95% ethanol for 40 s before counterstaining with Van Gieson solution (saturated aqueous picric acid:1% aqueous fuchsin at a ratio of 20:1) for 5 min. Excess stain was removed by blotting the slides with a filter paper. Finally, the slides were dehydrated in three changes of ethanol (95%, 100%, 100%), each for 10 sec, cleared in two changes of xylene (each for 10 s) and mounted on an automated cover slipper (Leica CV5030). 

Light Microscopy. Stained slides were air-dried and viewed using a light microscope (Zeiss Axiophot, West Germany) mounted with a Q Imaging Micropublisher 6 Camera. 

#### 2.3.5. Ultrastructure Analysis Using Transmission Electron Microscopy (TEM)

The TEM analysis was done following the protocol of the Massey Manawatu Imaging Centre (MMIC) (Sample Processing for TEM, unpublished) as described by Chian et al. [19]

Pre-Treatment. Raw and cooked treated samples from the central part of the foot and the adductor were cut into 10 × 3 × 3 mm pieces using a carbon steel surgical blade. Cut samples were immediately fixed in a modified Karnovsky fixative, followed by immersing in sodium cacodylate buffer. Post-fixing was done in 1% osmium tetroxide followed by washing and dehydration through a graded acetone series. Samples were then left overnight in a mixture of fresh resin and acetone (50:50) with constant stirring. The following morning, the mixture was discarded and replaced with 100% resin with catalyst and the sample was incubated for 8 h with constant stirring. Finally, the samples were embedded in 100% resin with the catalyst in a silicone mould and incubated in an oven at 60 °C for 48 h. Samples in resin blocks were removed from the mould and transferred to vials for cutting. 

Transmission Electron Microscopy. Blocks were cut into 80–100 nm sections using an ultramicrotome (Leica EM UC7). Sections were stretched with chloroform vapour using a Quick Coat G pen on a copper grid and stained for 4 min with saturated uranyl acetate in 50% ethanol. After this, samples were washed with 50% ethanol and Milli-Q water followed by staining with lead citrate for 4 min then again washed with Milli-Q water. Finally, micrographs were obtained using a Transmission Electron Microscope (FEI Technai G2 Spirit BioTwin, Czech Republic).

#### 2.3.6. Texture Analysis

The central part of the cooked pāua foot was used in determining the SSFV. The adductor was cut from the base, and the epipodia or lips were removed. Samples were cut into rectangular cross-sections (1 cm × 1 cm × 2 cm). The maximum peak force was measured using the TA.XT plus texture analyser (Stable Microsystems, Godalming, UK) calibrated accordingly before each batch of the test. Cut sections (a minimum of six pieces per pāua were analysed), were placed under a flat blade and cut perpendicular to the direction of the muscle fibres, using a load cell of 490.5 N. The test speed was 1 mm/s with a trigger force of 0.05 N.

#### 2.3.7. In Vitro Static Oral-Gastro-Small-Intestinal Digestion 

In vitro protein digestibility of raw and cooked samples (as mentioned in Section 2.3) were analysed using the method of Chian et al. [19]. Samples were comminuted to a 2 mm particle size using a kitchen electric meat mincer (Kenwood no. 1552) before analysis. 

Samples were taken after 2, 30 and 60 min of simulated gastric digestion, and 10, 60 and 120 min of simulated small intestinal digestion. Samples were immediately immersed in an ice bath and pepstatin A (Abcam, 12 µL per mL of digest) was added to stop the gastric digestion. Protease inhibitor (SigmaFast, Sigma Aldrich, Saint Louis, MO, USA) solution (250 µL per mL of digest) was added to stop the small-intestinal digestion. Samples were stored at −20 °C for further analysis. 

Preparation of the digests for further analysis. The digests were centrifuged at 13,000× *g* for 20 min at 2 °C. The supernatants were filtered through 0.45 µm polyvinylidene fluoride (PVDF) syringe filters, and the filtered samples were analysed for soluble nitrogen content and ninhydrin reactive amino nitrogen. For reduced-tricine sodium dodecyl sulphate-polyacrylamide gel electrophoresis (SDS-PAGE), the digests were homogenised prior to dilution with Milli-Q water to 4 mg of total protein per mL of the sample. Diluted digests were mixed with tricine sample buffer (M6250, Sigma Aldrich, Saint Louis, MO, USA) containing 0.26 mol/L β-mercaptoethanol (digest: buffer ration of 1:1) to a final concentration of 2 mg of total protein per mL of sample. 

Ninhydrin-reactive amino nitrogen. Digest supernatants were analysed for ninhydrin-reactive amino nitrogen (Free Amino N) using 2% ninhydrin reagent (N7285, Sigma-Aldrich, Saint Louis, MO, USA) as described by Moore [21] and Chian et al. [19].

Soluble nitrogen. The soluble nitrogen (%) in the supernatant of the digested samples after different digestion times was determined following the Kjeldahl method [22] as described by Kaur et al. [23].

Reduced-Tricine-sodium dodecyl sulphate- polyacrylamide gel electrophoresis (SDS-PAGE). Reduced tricine SDS-PAGE was carried out following the methods of Chian et al. [19] with some modifications. Twenty five (25) µL of samples (in buffer) and 10 µL of the pre-stained protein standard (Precision Plus protein™ dual Xtra, Bio-Rad Laboratories Pty Ltd., Auckland, New Zealand) were loaded into a precast gel (16.5% Criterion™ Tris-Tricine Gel, 18 well, 30 µL, Bio-Rad Laboratories Pty Ltd., New Zealand). SDS-PAGE was carried out at 125 V for about two hours. Afterwards, the gel was fixed in a solution of 40% methanol and 10% acetic acid in Milli-Q water for one hour. Staining was done with Coomassie Brilliant Blue R−250 (Bio-Rad Laboratories Pty Ltd., New Zealand) for 1 h followed by rinsing in Milli-Q water for 30 min. Images of the gels were obtained using a Gel Doc XR+ scanning densitometer (Bio-Rad Laboratories Pty Ltd., New Zealand). The bands were analysed using Image Lab™ software version 6.0.0.

### 2.4. Statistical Analysis

For each treatment, three individual pāua were analysed and each pāua was measured at least in triplicate. For texture analysis, at least six measurements per pāua were done. The data reported were analysed using Minitab 18 Statistical Software (Minitab Inc., State College, PA, USA). Analysis was done using One-way ANOVA using the Tukey method and a 95% confidence level. Results are expressed as means ± SD.

## 3. Results

### 3.1. Texture Measurements on Canned Pāua

**Figure 2:**
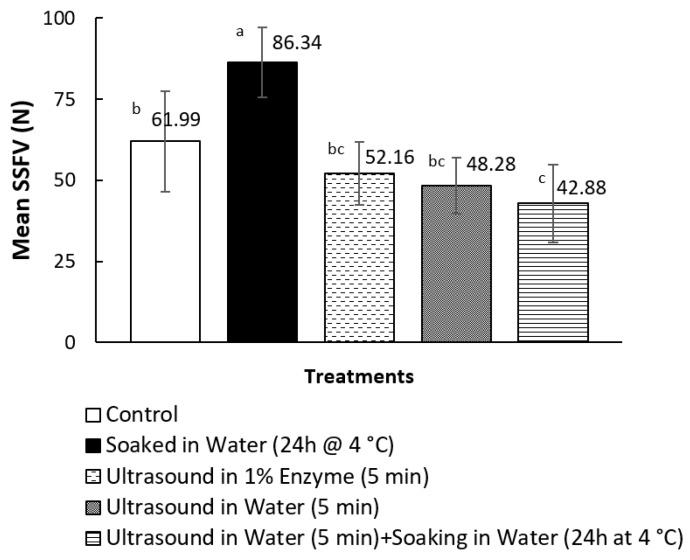
Effect of pre-treatments on the mean sliced shear force values (SSFV) of canned and cooked pāua. *N* = 3; means that do not share a letter are significantly different (*p* < 0.05).

None of the treatments mentioned in this study caused any undesirable changes in the appearance of the pāua. The samples did not show any damage, such as cracks or loss of the defined shape of the pāua lips. There was also no observed mushiness when the samples were cut with a knife. All cooked samples that had undergone ultrasonication as part of the pre-treatment yielded shear force values lower than the cooked control, as shown in Figure 2. However, only ultrasound pre-treatment in water for 5 min, followed by soaking in water for 24 h at 4 °C, led to significantly (*p* < 0.05) lower average SSFV than the cooked control. The treatment yielded the lowest SSFV, but this was not significantly different from ultrasound treatment for 5 min in water or 1% enzyme solution alone. On the other hand, soaking in water for 24 h alone, with no ultrasonication as pre-treatment, resulted in significantly higher SSFV than the cooked control.

### 3.2. Microscopy

#### 3.2.1. The Microstructure of Raw and Cooked Pāua Muscle 

**Figure 3:**
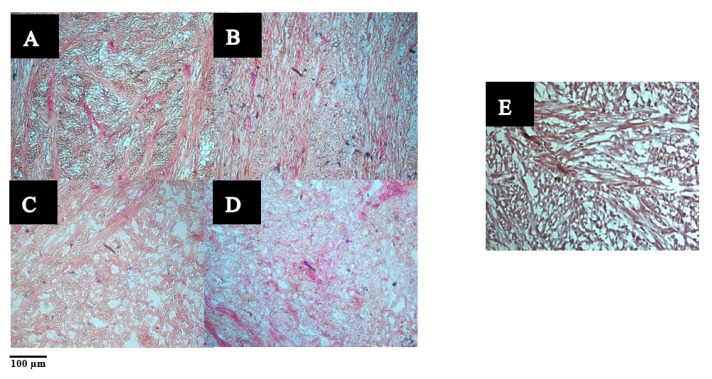
Light micrographs of pāua foot muscle tissues stained with Verhoeff-Van Gieson (VVG) staining. (**A**–**D**) Raw: (**A**) untreated, (**B**) ultrasound in water for 5 min followed by soaking in water at 4 °C for 24 h, (**C**) treated with (**C**) ultrasound in water for 5 min, (**D**) ultrasound for 5 min in 1% actinidin solution, (**E**) untreated cooked control.

Light micrographs showing the stained muscle fibres are shown in Figure 3. The collagen fibres appeared dark brown or red, while the myofibers appeared light brown in colour. The muscle fibres in the raw sample appeared compact with randomly arranged collagen fibres and myofibers, while the cooked samples exhibited wider spaces between the muscle fibres. Micrographs of the raw ultrasound- (in water or enzyme solution) treated samples (B–D) showed disintegration of the muscle fibres and exhibited a widening of the extracellular spaces. However, wider voids could be observed in the sample treated with ultrasound in water alone (C) than in the sample where ultrasonication was followed by soaking in water for 24 h (B). An average of 8 ± 2% increase in weight was noted for pāua samples after soaking. Thus, it is likely that water absorption had caused swelling and narrowed the gaps between the muscle fibres.

Muscle fibres of samples that had undergone ultrasonication appeared lighter in colour than their native form. In addition, muscle fibres after ultrasound treatment in 1% enzyme solution (D) appeared even lighter in intensity, and there seemed to be a dissolution of the myofibers due to enzyme action. 

#### 3.2.2. Ultrastructure of Raw and Cooked Pāua Muscle 

**Figure 4:**
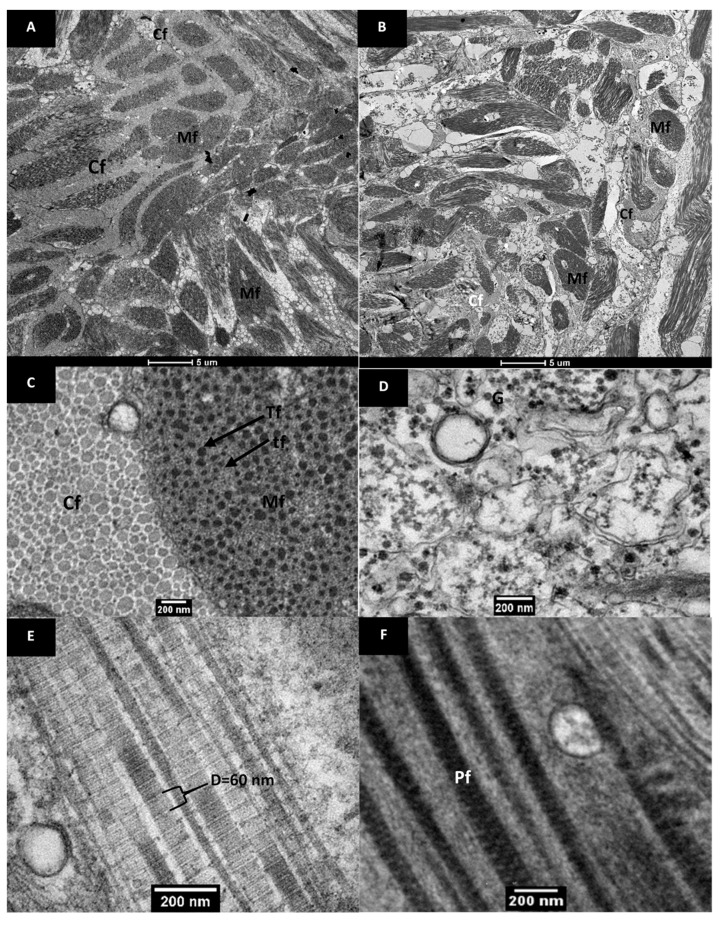
Transmission electron micrographs of raw pāua muscle tissue. Cross-sectional views of the (**A**) foot, and (**B**) adductor muscle part. (**C**–**F**) are from the foot muscle part. (**C**) shows a cross-sectional view of the collagen fibrils (CF) and the myofibrils (Mf) comprising the thick (Tf) and thin (tf) filaments, while (**D**) shows extracellular spaces with glycogen granules. (**E**) shows a longitudinal view of the collagen fibrils showing a banded structure and (**F**) is a longitudinal view of patterned thick filaments identified as paramyosin fibrils (Pf).

The transmission electron micrographs of native pāua muscle tissues are shown in Figure 4. Layers of collagen fibrils existed alongside the myofibrils, and these were visually more abundant in the foot (Figure 4A) than in the adductor (Figure 4B), which had more extracellular spaces. The results agreed well with previous reports on microstructure and chemical analysis, confirming the greater abundance of collagen in the foot muscle than in the adductor muscle for other species of abalone [24]. The collagen fibrils had diameters ranging from 20–150 nm (Figure 4C) and exhibited cross-striations with a periodicity of 40–60 nm (Figure 4E). These values are close to those reported by Olaechaea et al. [24] for *Haliotis discus*. 

The myofibrils appeared as long cylindrical cells that had slightly thickened centres and tapered towards the ends. The thin and thick filaments were arranged randomly, characteristic of smooth muscles, and were not aligned into the A and I bands that the striated muscles exhibited [25]. Invertebrate, and particularly mollusc, muscles can be categorised into three structure types: cross-striated, obliquely striated and smooth muscles [26,27]. Abalone is known to be composed of smooth muscles, and the random arrangement of myofibrils has been reported for other abalone species [7,24,28,29]. The random arrangement was also observed in the light micrographs (Figure 3). The thick filaments have diameters of about 20–80 nm, which is similar to those of turban shell (*Batillus cornutus*) reported by Ochiai et al. [30] Glycogen granules were observed in the extracellular spaces of the foot muscle (Figure 4D), which have also been reported by Olley and Thrower [17]. Patterned thick filaments were also observed in the foot muscle (Figure 4F). These filaments have been identified in previous studies as paramyosin fibrils [31], reported to be abundant in the thick filaments of the myofibrils of *Haliotis discus* in both the adductor and foot muscle [24]. 

**Figure 5:**
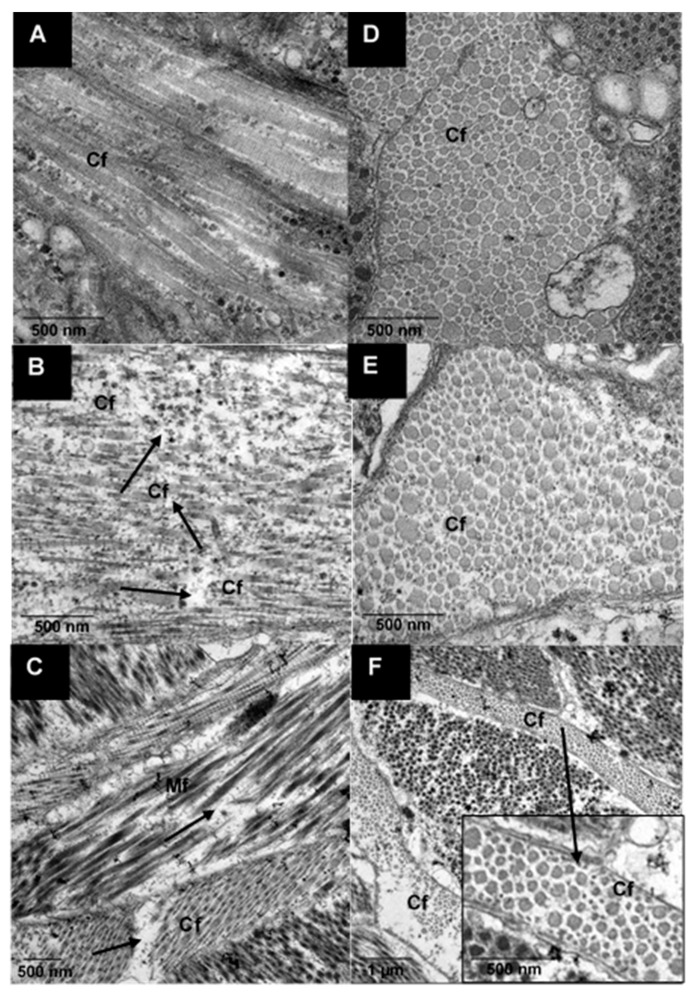
Transmission electron micrographs of raw pāua foot muscle tissue. Longitudinal (**A**,**B**) and transverse (**D**–**F**) views of the (**A**,**D**) untreated, (**B**,**E**) ultrasound treated in water for 5 min followed by soaking for 24 h and (**C**,**F**) ultrasound treated in 1% actinidin solution for 5 min; (Mf) myofibrils, (Cf) collagen fibrils.

The ultrastructure of samples, as affected by ultrasound treatments, is shown in Figure 5. Ultrasound treatment caused visible fragmentation of the collagen fibrils (marked with arrows) as shown in the TEM images of lateral sections (Figure 5A–C). This fragmentation could be observed in all ultrasound-treated samples, whether in water or in actinidin enzyme solution. From the cross-sectional images, it could be observed that the gap between collagen fibrils had widened and this was more pronounced in enzyme-treated samples (Figure 5F). Additionally, fragmentation of the myofibrils could be seen in the sample that was ultrasonicated in the enzyme solution (Figure 5C). Denaturation of collagenous fibres as a result of ultrasonication of beef meat steaks has been reported in a previous study [32]. Moreover, actinidin has been reported to cause tenderness in meat by weakening the connective tissues and structural damage to the Z disc of the myofibril [14,33].

**Figure 6:**
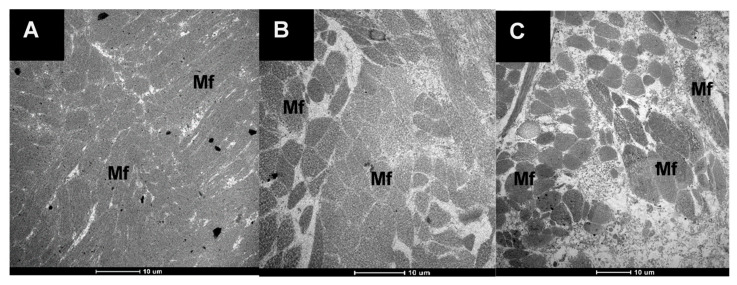
Transmission electron micrographs of the muscle tissues of pāua that were canned and cooked in a water retort at 116 °C for 30 min. (**A**) untreated (control), (**B**) ultrasonicated in water for 5 min followed by soaking for 24 h, and (**C**) ultrasonicated in 1% actinidin solution for 5 min; (Mf) myofibrils.

The ultrastructure of the cooked pāua muscle samples is shown in Figure 6. Cooked muscle tissues of untreated pāua and ultrasound-treated samples could be easily differentiated by the size of the gaps between the aggregated myofibrils. The untreated sample exhibited a very compact structure with very thin gaps. Previous studies reported large extracellular spaces caused by the destruction of some of the myofibrils when abalone (*Haliotis discus*) was heated at 100 °C for 60 min [5]. However, in this study, control samples, canned and cooked at 116 °C for 30 min, exhibited small voids in between the myofibrils. The compact structure of the myofibrils in untreated cooked samples could be caused by aggregation of the muscle proteins due to a very high cooking temperature. 

Ultrasound-treated cooked samples had large spaces in between myofibrils, which were more pronounced in the samples ultrasonicated in enzyme solution. The differences in the extracellular spaces between the two cooked samples seemed to correlate with the degree of fragmentation in the myofibrils observed in the raw samples. 

### 3.3. pH, Cook Loss, Total Nitrogen Content and Thermal Properties

The pH, total nitrogen content (%) and cook loss (%) of raw and cooked control, and cooked ultrasound-treated (5 min in water followed by soaking for 24 h at 4 °C) pāua are shown in Table 1. The pH of the raw samples was similar to the pH recommended (6.2–6.4) for canning abalone and was well above the isoelectric point (4.8–5.2) previously reported for *Haliotis discus* meat [34]. A high pH before canning is essential in reducing cook loss. A pH near the isoelectric point may cause shrinkage of the myofibrillar proteins and consequent reduction of the water holding capacity of the meat [35], which will lead to toughness. From Table 1, it can be observed that for all samples, cooking resulted in a significant increase (*p <* 0.001) in pH from the raw control, but the differences between cooked control and ultrasound-treated cooked samples were not significant. However, there was a significant difference in the SSFV of the two samples (Figure 2, Section 3.1). Previous research on bovine meat has also reported an increase in pH after cooking [36,37]. Hamm and Deatherage [38] have reported the small increase in pH after cooking of meat to be due to a decrease in acidic groups in the meat proteins [39]. The pH of the cooked samples obtained from this study was comparable to the previously reported pH values for canned abalone [35]. All ultrasound pre-treatments resulted in lower thermal denaturation temperatures (as observed using DSC) than the control, although the difference was not significant (*p <* 0.05, data not shown).

There was also no significant difference in cook loss (%) among the control and ultrasound pre-treated samples. Cooking led to a significant decrease in the total nitrogen content (%) of the abalone samples, which could be due to the loss of soluble protein components during cooking as drip loss. However, the total nitrogen content (%) was significantly lower (*p <* 0.001) for the cooked control samples than for the cooked ultrasound pre-treated samples. 

### 3.4. In Vitro Gastro-Small Intestinal Digestibility 

#### 3.4.1. Ninhydrin Assay 

**Figure 7:**
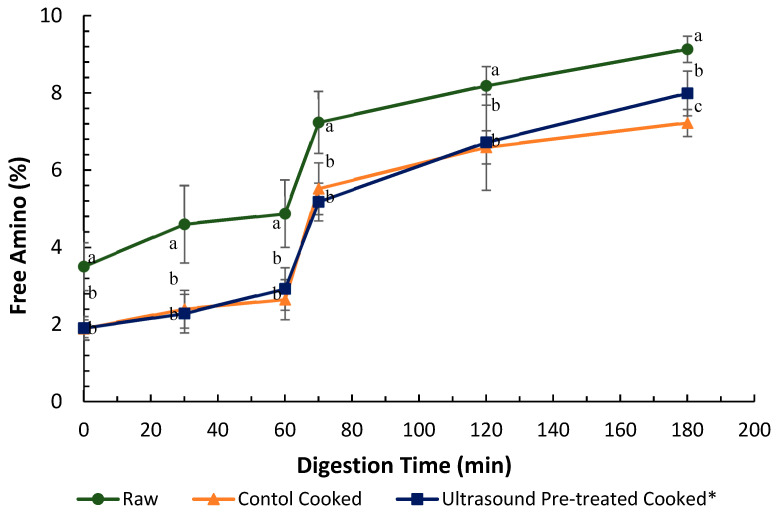
In vitro protein digestibility of raw 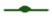 and cooked (canned) control 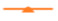 and cooked ultrasound pre-treated 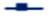 pāua determined based on ninhydrin-reactive free amino N released during gastro-small intestinal digestion (*n* = 3. Ultrasound treatment was done in water for 5 min followed by soaking in water for 24 h at 4 °C. Digestion times 0, 30 and 60 min represent 2, 30 and 60 min of digestion in the gastric phase following 2 min of oral digestion phase; 70, 120 and 180 represent 10, 60 and 120 min of digestion in the small-intestinal phase following 60 min of gastric and 2 min of oral digestion phases. Different letters in the same column represent a significant difference (*p* < 0.05).

The in vitro protein digestibility of raw control, cooked control and pre-treated (ultrasound in water for 5 min followed by soaking in water for 24 h at 4 °C) pāua was determined based on the ninhydrin-reactive amino nitrogen released at specific digestion time points (Figure 7). The amounts of free amino N (%) detected in samples digested without any added digestive enzymes after 180 min of digestion time were comparable and not significantly different (data not shown) from digested samples after 2 min of gastric digestion, which had followed 2 min of oral digestion (Figure 7). Protein hydrolysis does not typically occur in the oral phase of digestion because there is no proteolytic activity in saliva [39]. 

It is apparent from Figure 7 that there was a minimal increase in free amino groups from 0 to 60 min of gastric digestion, but a steep rise in free amino nitrogen could be observed within the first 10 min of small intestinal digestion. 

Overall, the raw samples showed significantly higher free amino N than the cooked samples throughout the digestion. The difference in free amino N is consistent with the loss of soluble protein (or peptides and free amino acids) due to cook loss during cooking (Section 3.3). Ultrasound-treated cooked samples showed significantly (*p <* 0.001) higher protein digestibility (than the cooked control) after 180 min of in vitro digestion. 

Consistent with the ninhydrin assay results, raw samples had significantly higher soluble nitrogen at the start of the digestion than the cooked samples (Table 2). This observation is also consistent with the observed decrease in total nitrogen (Section 3.4) in cooked abalone samples. There was a sharp increase in soluble nitrogen during the first 10 min of small-intestinal digestion. However, the soluble N contents did not differ significantly at the end of the gastric phase or the end of total digestion, among the controls and the treated samples. 

#### 3.4.2. SDS-PAGE 

**Figure 8:**
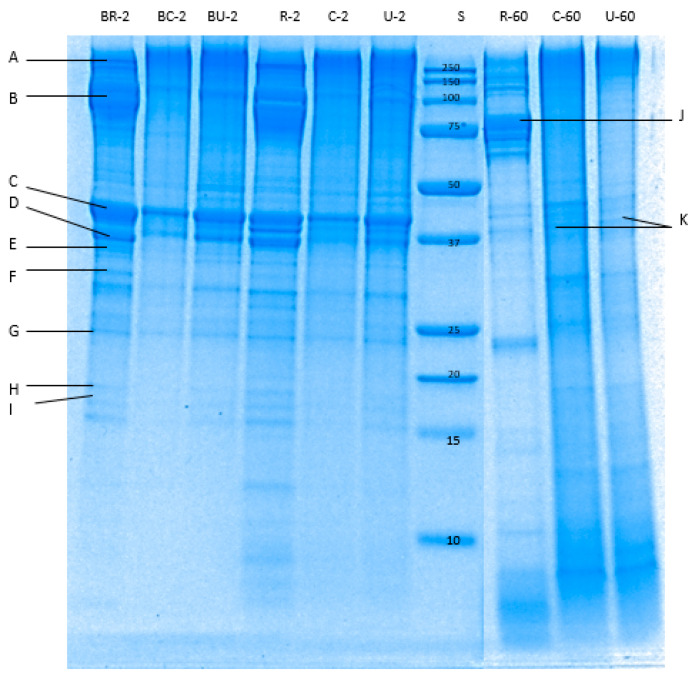
Tricine sodium dodecyl sulphate-polyacrylamide gel electrophoresis (SDS-PAGE) electrophoretogram showing protein profile of the digested pāua samples after 2 or 60 min of gastric digestion (following 2 min oral digestion). S, standard; R, raw control; C, cooked control (no pre-treatment prior to canning); U, cooked ultrasound pre-treated; BR, BC and BU, are digested without enzymes; 2 and 60 are the digestion times. A–K bands correspond respectively to myosin-heavy chain (>200 kDa); paramyosin (100 kDa); actin (45 kDa); tropomyosin-β chain (40 kDa); troponin T (36 kDa); tropomyosin-α chain (33 kDa); myosin-light chain (26 kDa); troponin C (20 kDa); myosin-light chain (17 kDa); 77 kDa peptide; tropomyosin (35–38 kDa). Ultrasound treatment was done in water for 5 min followed by soaking in water for 24 h at 4 °C prior to canning.

**Figure 9:**
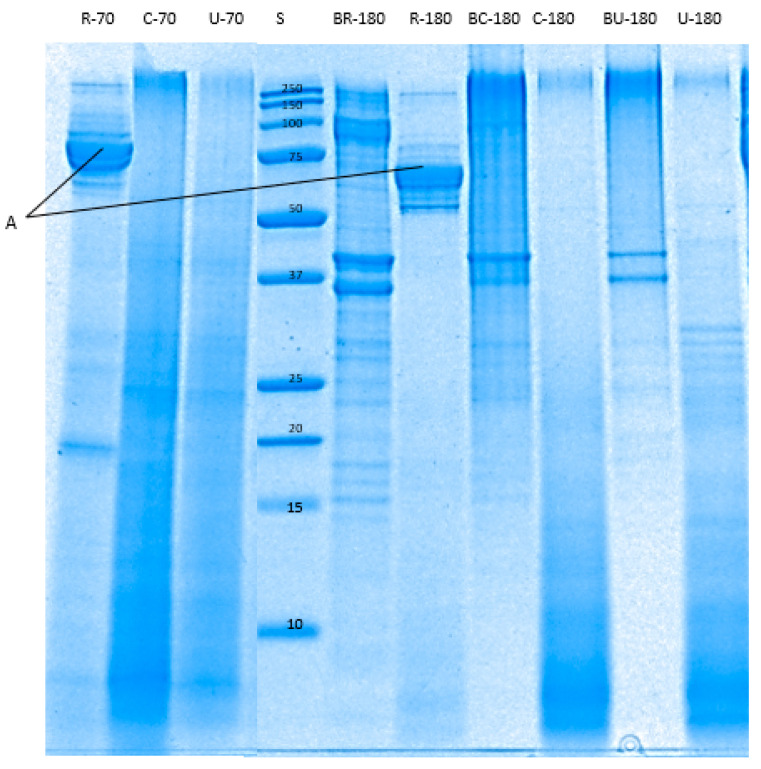
Tricine SDS-PAGE electrophoretogram showing protein profile of the digested pāua samples after 10 or 120 min of small-intestinal digestion. S, standard; R, raw control; C, cooked control (no pre-treatment prior to canning); U, cooked ultrasound pre-treated; BR, BC and BU, are digested without enzymes; 70 and 180 are the digestion times (following 60 min gastric and 2 min oral digestion), in min. A, 75–79 kDa peptides. Ultrasound treatment was done in water for 5 min followed by soaking in water for 24 h at 4 °C prior to canning.

The enzymatic breakdown of pāua proteins during in vitro digestion in gastric (Figure 8) and small intestine (Figure 9) phases were determined through reduced tricine-SDS-PAGE. Raw pāua digested without any added digestive enzymes (Figure 8) and showed bands corresponding to myosin heavy chains, paramyosin, actin and tropomyosin, among others, which are similar to those reported by Zhu et al. [5] for other species of abalone (*Haliotis discus*). These protein bands were also observed in the cooked samples regardless of treatment, except for the band corresponding to paramyosin. Tropomyosin has been reported to remain after the cooking of abalone [40], which is also the case with the pāua samples (Figure 8K). High-molecular-weight (HMW) aggregates (MW 250 kDa) could also be observed from the electrophoretograms, and these were more intense in control-cooked samples than in cooked ultrasound pre-treated and raw samples. After 60 min of gastric digestion, cooked and raw samples exhibited different susceptibility to hydrolysis by pepsin. Protein aggregates (MW > 250 kDa) were hydrolysed by pepsin to a greater extent in raw than in cooked samples. A new group of peptides (MW 77 and 75 kDa) that could be observed at the end of small-intestinal digestion could be observed in the raw sample (Figure 8J and Figure 9A) which was not present in the cooked samples. Digestion by the pancreatic enzymes was marked by a rapid decrease in the intensity of the bands corresponding to HMW aggregates (250 kDa) and large peptides (MW > 100 kDa) after 10 min. Bands with MW between 44 and 12 kDa, and MW < 10 kDa could be observed after 10 min of small-intestinal digestion of both cooked samples. These bands might have been formed from the hydrolysed HMW peptides or from pepsin hydrolysates that were not yet digested by pancreatin after 10 min. LMW peptides (MW < 25 kDa) in the pepsin hydrolysates of raw samples were completely digested after 180 min of gastro-small intestinal digestion while protein bands with MW < 37 kDa remained in cooked samples. However, bands with MW 55 to 92 kDa persisted in raw samples even after 180 min of gastro-small intestinal digestion. After about 180 min (2 min oral, 60 min gastric and 120 min small-intestine) of digestion, very faint bands of HMW aggregates remained in all samples, with different intensities in the following order: R−180 < U−180 < C−180. Cooking did not seem to affect the protein breakdown of actin and tropomyosin.

## 4. Discussion

The objective of this study was to identify processing techniques that will lead to the tenderisation of canned pāua and to obtain information on the in vitro protein digestibility of the product. Some preliminary experimentation was performed using ultrasonication and actinidin enzyme solution, individually and in combination. The cooked pāua appearance was evaluated based on industry standards for canned pāua [41]. The epipodium should retain its defined shape, and the adductor should be smooth without any damage or cracking. The treatments presented in this study were selected as they led to lower SSFVs than the control canned commercial sample but did not cause any undesirable effects on the appearance of the canned paua. Automated injection of enzyme solution and soaking pāua in higher enzyme concentration (5% *w*/*v*) were eliminated from further studies, despite their tenderising potentials, due to their adverse effects on the appearance of the cooked pāua. These treatments led to cracks on the surface of the pāua and caused sliminess and loss of definition on the pāua lip, which were deemed unacceptable. 

Ultrasound pre-treatment in water for 5 min followed by soaking in water for 24 h at 4 °C, yielded the lowest average SSFV (42.88 N) and the samples were on average 31% more tender than the canned control (61.99 N). Increased tenderness caused by low frequency, high-intensity ultrasound applied for a sufficient time, has previously been reported in several studies on beef muscles [42,43,44]. The average SSFV exhibited by the above-mentioned sample was within the reported acceptable slice shear force reported by previous studies. Dong et al. [6] reported highest sensory acceptability in terms of structure (meat integrity), hardness and elasticity for cooked abalone that yielded shear force values of 36.52 ± 5.19 N. Shear force (Warner-Bratzler) values have been strongly positively correlated with the sensory hardness and chewiness (product of hardness, elasticity and cohesiveness) of abalone [3]. In a different study, Zhu et al. [5] recommended a heating temperature for cooking abalone that yielded an average SSFV of 56.47 ± 9.79 N. There were differences in the methods for shear force measurements, such as sample thickness and type of blade, which could have affected the values. 

The commercial canned samples had comparable SSFV (68.54 ± 6.58 N) with canned control (61.99 ± 15.40 N), which means that the treatments have shown potential in improving the tenderness of canned pāua. However, the commercial samples are usually cleaned in brine, bleached and were cooked in a medium that has salt and sugar, which could affect abalone meat tenderness. Ultrasound treatment may be applied during the cleaning steps prior to canning, but industrial practices use salt solution for this process [45], which may have an influence on abalone tenderness. Further investigations on these interactions are needed.

Changes in the structure of the muscle fibres during cooking related well to the texture measurements. Significant disruption of muscle fibres and a widening of extracellular spaces was seen in ultrasound-treated samples and this could be responsible for their lower SSFVs compared to the control. Although the ultrastructure of the cooked sample pre-treated by ultrasound in enzyme showed much-staggered myofibrils, its mean SSFV was higher (but not significantly, *p* > 0.05) than the mean SSFV of samples ultrasonicated in water. Cooking led to a narrowing of the extracellular spaces, with the structure becoming more compact than the raw uncooked pāua. This could be due to loss of water as cook loss. Similar results have been reported by Kaur et al. [46] after cooking at 100 °C for beef muscles. Hatae and co-authors [8] reported an increase in the total amount of free amino acids and oligopeptides in the meat drips after extended cooking, and this was attributed to the shrinking of the meat that squeezed out these components [8]. Shrinkage of the myofibrillar proteins could be observed in the TEM images of cooked pāua (Figure 6, Section 3.2.2). New inter- and intra-protein interactions arising from exposure of hydrophobic areas, along with shrinkage of some proteins such as titin during cooking, can result in a compact protein structure [47]. Although the pāua samples in the current study were cooked for only 30 min, the temperature used (116 °C) was substantially higher than that used in previous studies. Other than the cooking time, the increased temperature has also been implicated in the reduction of soluble protein in cooked meat [48]. In agreement with the previous literature reports, cooking of pāua also led to a change in the protein breakdown pattern as observed through SDS-PAGE along with a significant reduction in soluble nitrogen during simulated gastric digestion. There was a relatively small amount of gastric hydrolysis as observed from free amino N despite the loss of some intact protein as seen in the gels. Pepsin preferentially cleaves adjacent to aromatic amino acids (e.g., phenylalanine, tyrosine and tryptophan) [49], but it does not hydrolyse peptide bonds adjacent to valine, alanine or glycine [50]. It converts proteins to smaller peptides and is reportedly responsible for less than 20% of protein hydrolysis during gastro-small intestinal digestion [51]. Additionally, the low pH (3) of the simulated gastric fluid might have induced the formation of a matrix of coagulated proteins that were insoluble and resistant to further pepsin hydrolysis, thus explaining the lower free amino N values during the gastric digestion. 

A decrease in protein digestibility has been reported after cooking by Santé-Lhoutellier et al. [52] and Kaur et al. [46] for pork and beef meat. In contrast, Shi et al. [53] stated that heating did not affect the digestibility of proteins in abalone, *Haliotis discus*. The paper did not indicate the temperature and time combination used in heating. A negative and strong correlation with pepsin activity has been reported for carbonyl group formation and aggregation that was induced by cooking [52]. In the present study, pāua samples were cooked at 116 °C for 30 min, and protein aggregation was observed in the electron micrographs, however, the differences observed in free amino N among raw and cooked samples are consistent with the loss of soluble protein components during cooking. SDS-PAGE results showed a group of peptides (MW 77 and 75 kDa, Figure 8J and Figure 9A) that are seen in the raw pāua digest during gastric digestion, that were resistant to further digestion and could be observed at the end of small-intestinal digestion, were not seen in the cooked samples. Similar bands were observed during the gastric digestion of raw beef meat by Kaur et al. [46] They reported these peptides to be the hydrolysis products of a myosin-heavy chain (MHC, 220 kDa). Meat is oxidised and denatured upon cooking, leading to MHC’s breakdown into even smaller MW peptides, which could be the reason for not observing these bands in the cooked meat digests. Bands corresponding to HMW protein aggregates could still be observed in all the digests at the end of digestion, with their higher intensities observed for the control-cooked digest. A recent study on the digestion of the myofibrillar protein fractions of raw abalone, *Haliotis discus,* showed some 60–200 kDa protein bands that were seen after 120 min of pepsin-trypsin digestion [53]. The combination of thermal treatment and ultrasonication of cowpea proteins has been reported to contribute to protein unfolding, which has been suggested to lead to increased hydrolysis [54]. This could explain the higher protein digestibility of ultrasound-treated cooked pāua compared with cooked pāua muscle.

## 5. Conclusions

In general, ultrasound pre-treatment led to increased tenderness of cooked pāua. Particularly, ultrasound for 5 min in water followed by soaking in water at 4 °C for 24 h exhibited the greatest potential in texture improvement of cooked pāua meat. It should be noted that the SSFV of ultrasound-treated cooked samples (followed by soaking in water) were significantly lower than control but did not exhibit significant differences from other ultrasound-treated samples, including those that did not undergo soaking for 24 h. The observed increased tenderness due to ultrasound pre-treatment may be linked to the disintegration of muscle fibres, as observed in the light micrographs, and fragmentation of collagenous fibrils observed using TEM of raw pre-treated samples. This was more pronounced with the sample sonicated in 1% actinidin enzyme solution, which also caused fragmentation of the myofibrils. The observed disruption of muscle fibrils in the raw treated samples was found to correlate with the size of extracellular spaces observed in the cooked samples. Untreated cooked pāua exhibited very compact myofibers. On the other hand, ultrasound pre-treated samples had wider gaps; pāua sonicated in enzyme solution showed larger spaces between myofibers than those sonicated in water. Raw pāua exhibited significantly higher (*p <* 0.05) free amino N values during in vitro digestion and also protein breakdown as observed through SDS-PAGE than cooked control and ultrasound pre-treated samples (ultrasound in water for 5 min followed by soaking for 24 h at 4 °C. However the digestibility in terms of total free amino N release during digestion and the overall protein breakdown of ultrasound-treated cooked samples was significantly higher than the cooked control. This suggests that digestibility properties of pāua are also influenced by the above-discussed microstructural and ultrastructural changes induced by cooking and the ultrasound treatment.

## Figures and Tables

**Table 1:** pH, total nitrogen content (%), and cook loss (%) for control, and ultrasound pre-treated cooked pāua.

Samples	pH of Abalone	Total Nitrogen (%) of Abalone	Cook Loss (%)
Control raw	6.05 ± 0.13 ^b^	3.06 ± 0.14 ^a^	-
Control cooked	6.63 ± 0.02 ^a^	2.49 ± 0.24 ^c^	6.55 ± 0.23 ^a^
Ultrasound pre-treated cooked	6.68 ± 0.10 ^a^	2.72 ± 0.08 ^b^	9.14 ± 2.89 ^a^
*p* value	<0.001	<0.001	0.196

Ultrasound treatment was done in water for 5 min followed by soaking in water for 24 h at 4 °C. Pāua were canned and cooked in water retort at 116 °C for 30 min. Results are expressed as means (*n* = 3) ± SD; different letters in the same column represent a significant difference (*p <* 0.05).

**Table 2:** Soluble nitrogen (%) of raw and cooked control, and cooked ultrasound pre-treated pāua after 2, 30, 60, 70, 120 and 180 min of in vitro oral and gastro-small intestinal digestion.

Digestion Time (min)	Soluble Nitrogen (%)		
	Control Raw	Control Cooked	Ultrasound Cooked
2	34.09 ± 3.4 ^a^	18.06 ± 5.10 ^b^	15.23 ± 1.07 ^b^
30	46.84 ±5.35 ^a^	33.90 ± 3.73 ^ab^	25.49 ± 9.83 ^b^
60	52.79 ± 5.82 ^a^	42.03 ± 7.98 ^a^	36.61 ± 10.57 ^a^
70	66.65 ± 4.73 ^a^	62.10 ± 4.89 ^ab^	50.97 ± 6.96 ^b^
120	72.49 ± 6.83 ^a^	75.82 ± 8.38 ^a^	68.15 ± 3.46 ^a^
180	84.72 ± 11.82 ^a^	76.21 ± 6.52 ^a^	74.73 ± 4.13 ^a^

Ultrasound treatment was done in water for 5 min followed by soaking in water for 24 h at 4 °C. Results are expressed as means (*n* = 3) ± SD; different letters in the same row represent a significant difference (*p <* 0.05).
